# Prediction of poststroke cognitive impairment based on the systemic inflammatory response index

**DOI:** 10.1002/brb3.3372

**Published:** 2024-01-06

**Authors:** Min Chu, Yunhe Luo, Daosheng Wang, Zhuohang Liu, Huicong Niu, Xuechun Wu, Yong Wang, Jixian Lin, Qiang Wang, Jing Zhao

**Affiliations:** ^1^ Department of Neurology Minhang Hospital Fudan University Shanghai China; ^2^ Department of Neurosurgery Minhang Hospital Fudan University Shanghai China; ^3^ Department of Cardiothoracic Surgery Zhoupu Hospital Affiliated to Shanghai Medical College of Health Shanghai China

**Keywords:** acute ischemic stroke, inflammation, nomogram, poststroke cognitive impairment, systemic inflammatory response index

## Abstract

**Background:**

Poststroke cognitive impairment (PSCI) is a prevalent complication among stroke survivors. Although the systemic inflammatory response index (SIRI) has been shown to be a reliable predictor of a variety of inflammatory diseases, the association between the SIRI and PSCI is still unclear. Therefore, the purpose of this study was to investigate the relationship between SIRI and PSCI, and to design a nomogram to predict the risk of PSCI in acute ischemic stroke (AIS) patients.

**Methods:**

A total of 1342 patients with AIS were included in the study. Using the Mini‐Mental State Examination scale, patients were separated into PSCI and non‐PSCI groups within 2 weeks of stroke. Clinical data and SIRI values were compared between the groups. We developed the optimal nomogram for predicting PSCI using multivariate logistic regression. Finally, the nomogram was validated using the receiver operating characteristic curve, calibration curve, and decision curve analysis (DCA).

**Results:**

In total, 690 (51.4%) patients were diagnosed with PSCI. After adjusting for potential confounders, the SIRI (OR = 1.226, OR: 1.095–1.373, *p* < .001) was shown to be an independent risk factor for PSCI in the logistic regression analysis. The nomogram based on patient gender, age, admission National Institutes of Health Stroke Scale scores, education, diabetes mellitus, and SIRI had good discriminative ability with an area under the curve (AUC) of 0.716. The calibration curve and Hosmer–Lemeshow test revealed excellent predictive accuracy for the nomogram. Finally, the DCA showed the good clinical utility of the model.

**Conclusion:**

Increased SIRI on admission is correlated with PSCI, and the nomogram built with SIRI as one of the predictors can help identify PSCI early.

## Graphical abstract

Using data from 1342 patients with ischemic stroke, this study identifies 6 predictors for post‐stroke cognitive impairment, including age, gender, years of education, admission NIHSS scores, diabetes mellitus and systemic inflammatory response index. A nomogram based on these factors was established for predicting post‐stroke cognitive impairment. It may be helpful for therapeutic decision‐making and patients surveillance.

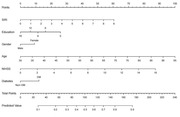

## 1 INTRODUCTION

Stroke is one of the leading causes of severe disability and mortality around the world (Avenatti et al., 2023). Cognitive impairment, a common complication of stroke, imposes an enormous financial burden on the families of stroke survivors (Boutros et al., 2023). Poststroke cognitive impairment (PSCI) has reportedly been related to a poor prognosis, including severe disability, mortality, stroke recurrence, and poor life quality (Kwon et al., 2020; Levine et al., 2015; Mijajlović et al., 2017; Rajan et al., 2014). The American Heart Association/American Stroke Association recommends that all stroke patients have a cognitive function evaluation before discharge (Winstein et al., 2016). Previous research found that using the Mini‐Mental State Examination (MMSE) within 2 weeks following a stroke has a predictive effect for PSCI 3–6 months later (Salvadori et al., 2013; Zhu et al., 2020; Zietemann et al., 2018). Nevertheless, there is no identified biomarker that can accurately predict the occurrence of cognitive impairment during the acute phase of a stroke, limiting the initiation of early intervention. As a result, there is an urgent need to explore novel and reliable indicators capable of detecting patients at risk of PSCI, especially in the acute phase following a stroke.

A growing body of evidence has suggested that inflammation plays a significant part in the neurodegenerative cascades of cognitive impairment (Shen et al., 2019). Peripheral inflammatory mediators have the ability to penetrate the blood–brain barrier (BBB), causing central inflammatory processes and neurodegeneration that impair cognitive function (Monje et al., 2003). Nerve regeneration, synaptic plasticity, and neurotransmission are further interfered by central inflammation, which results in brain atrophy and cognitive impairment (Perry et al., 2007). According to previous studies, inflammatory indicators, such as C‐reactive protein (CRP), interleukin‐6 (IL‐6), and interleukin‐8 (IL‐8), have been identified as important signal molecules in inflammation, which are closely associated with PSCI (Grigolashvili & Mustafina, 2021; Guo et al., 2018; Wang et al., 2022). Recently, the systemic inflammatory response index (SIRI), a new indicator of systemic inflammation, has been proposed as a combination of neutrophil, monocyte, and lymphocyte counts. SIRI has been shown to effectively reflect inflammatory status and predict outcomes in a variety of disorders, including aneurysmal subarachnoid hemorrhage and various malignancies (Zhang et al., 2020; Zhou et al., 2021). However, it is uncertain if SIRI is associated with PSCI.

We hypothesized that acute ischemic stroke (AIS) patients with elevated inflammation would be more likely to experience cognitive impairment after the stroke. Hence, this study aimed to investigate the relationship between SIRI and cognitive outcomes in the acute phase of AIS.

## 2 MATERIALS AND METHODS

### 2.1 Study design and participants

From January 2018 to December 2022, patients with AIS were recruited consecutively at the Minhang Hospital of Fudan University. The inclusion criteria were as follows: (i) aged ≥18 years; (ii) were diagnosed as AIS by magnetic resonance imaging (MRI) or computed tomography (CT); (iii) were willing to cooperate in completing the MMSE assessment and other relevant tests. The exclusion criteria were as follows: (i) acute infection, cancer, hematological disease, severe hepatic or renal diseases, as well as glucocorticoid or antibiotic use that affect inflammatory indicators; (ii) history of pre‐SCI; (iii) unable to complete cognitive assessment due to aphasia, vision impairment, hearing difficulty, and severe limb paralysis; (iv) history of central nervous system disorders; and (v) lack of available data. This study was reviewed and approved by the Institutional Ethics Committee of Minhang Hospital of Fudan University (ref. no. 2022‐044‐01K).

### 2.2 Data collection

We collected general clinical information during hospitalization, such as age, sex, years of education, medical history, and systolic and diastolic blood pressure measured at admission. The National Institutes of Health Stroke Scale (NIHSS) was used to assess the severity of stroke at admission, and the Trial of ORG 10172 in Acute Stroke Treatment criteria was applied to define stroke subtypes. All patients performed MRI or CT scans within 72 h of admission.

### 2.3 Evaluation of cognitive function

The cognitive function was evaluated by experienced neurologists in the early phase (within 2 weeks) after the onset of the stroke, when symptoms of cerebral infarction were relatively stable. The MMSE was used to assess overall cognitive function, with a total score of 30. Patients with an MMSE score ≤24 points were considered to have cognitive impairment (Chausson et al., 2010; Zhou et al., 2021).

### 2.4 Laboratory tests

Fasting blood samples were collected with EDTA‐K2 anticoagulant vacuum tube after at least 8 h of fasting within 24 h of hospital admission. All serum and plasma samples were separated and immediately frozen at −80°C until laboratory testing. Routine laboratory determinations (blood glucose, blood lipids, etc.) were performed for all enrolled patients at Minhang Hospital. Within 30 min after reversing evenly, complete blood counts, including white blood cells (WBCs), neutrophils (parameter: Neu#), lymphocytes (parameter: Lym#), monocytes (parameter: Mon#), and other parameters, were carried out using an automatic Mindray CAL 8000 blood cell analyzer (BC‐6800). Laboratory technicians who performed these measurements were blind to the clinical characteristics and outcomes of the study participants. We calculated the SIRI as follows: SIRI = NEUT × Mo/TLC, where NEUT is the neutrophil count, Mo is the monocyte count, and TLC is the total lymphocyte count.

### 2.5 Statistical analysis

Statistical analyses were performed using SPSS (version 26.0, IBM Corp) and R version 4.2.1. All patients were separated into PSCI and non‐PSCI groups. Non‐normally distributed continuous variables were represented as median (interquartile range) and compared using the Mann–Whitney *U* test. Normally distributed continuous variables were reported as mean ± SD and compared using the *t*‐test. All the independent variables we included were not normally distributed, so we used the Mann–Whitney *U* test to compare the two groups. Categorical variables were expressed numerically (percentages, %) and compared using the chi‐squared or Fisher's exact test. *p* < .05 (two‐sided) was considered to be statistically significant. The results are presented in Tables 1 and 2. Multivariate logistic regression models included variables with a univariate analysis result of *p* < .05 to obtain independent risk factors. Subsequently, we developed a nomogram for poor prognosis based on the independent risk factors, which were carried out by the “rms” package in RStudio (Zhang & Kattan, 2017). The nomogram assessed the model's discriminative ability by using the receiver operating characteristic (ROC) curve, which was carried out by the “pROC” package in RStudio. Calibration was evaluated using the Hosmer–Lemeshow test and calibration curve with a bootstrap of 1000 resamples, which were carried out by the “cal” and “p.hoslem” functions in RStudio. Furthermore, decision curve analysis (DCA), which was carried out by calculating the net benefit at various threshold risks of the model using the package “rmda,” was used to assess the nomogram's clinical usefulness. All figures were created with R version 4.2.1.

**TABLE 1:** Comparison of baseline characteristics between the poststroke cognitive impairment (PSCI) and non‐PSCI groups.

	Total (*n* = 1342)	Non‐PSCI (*n* = 652)	PSCI (*n* = 690)	*p*‐Value
Age (years)	68 (60–76)	65 (56–72)	71 (64–79)	<.001
Gender—female, *n* (%)	452 (33.7%)	178 (27.3%)	274 (39.7%)	<.001
Education (years)	9 (6–12)	9 (6–12)	9 (6–12)	<.001
Admission NIHSS scores	3 (1–4)	2 (1–4)	3 (2–5)	<.001
Systolic pressure (mmHg)	140.00 (130.00–151.25)	140.00 (130.00–150.00)	140.00 (130.00‐156.00)	.145
Diastolic pressure (mmHg)	80.00 (75.00–90.00)	80.00 (75.00–90.00)	80.00 (73.75‐89.25)	.589
**Vascular risk factors**				
Hypertension, *n* (%)	792 (59.0%)	369 (56.6%)	423 (61.3%)	.080
Diabetes mellitus, *n* (%)	378 (28.2%)	153 (23.5%)	225 (32.6%)	<.001
Atrial fibrillation, *n* (%)	93 (6.9%)	29 (4.4%)	64 (9.3%)	.001
Smoking history, *n* (%)	332 (24.7%)	192 (29.4%)	140 (20.3%)	<.001
Drinking history, *n* (%)	146 (10.9%)	77 (11.8%)	69 (10.0%)	.287
**Infarction location**				.824
Anterior circulation, *n* (%)	893 (66.5%)	436 (66.9%)	457 (66.2%)	
Posterior circulation, *n* (%)	327 (24.4%)	160 (24.5%)	167 (24.2%)	
Both, *n* (%)	122 (9.1%)	56 (8.6%)	66 (9.6%)	
**Stroke subtype**				.105
LAA*, n* (%)	823 (61.3%)	394 (60.4%)	429 (62.2%)	
CE*, n* (%)	106 (7.9%)	42 (6.4%)	64 (9.3%)	
SAO*, n (%)*	366 (27.3%)	190 (29.1%)	176 (25.5%)	
SOE*, n* (%)	4 (0.3%)	1 (0.2%)	3 (0.4%)	
SUE*, n* (%)	43 (3.2%)	25 (3.8%)	18 (2.6%)	
**Laboratory tests**				
WBC counts (10^9^/L)	6.63 (5.55–8.02)	6.63 (5.47–7.94)	6.65 (5.62–8.08)	.201
Platelet counts (10^9^/L)	206.00 (169.00–245.00)	207.00 (170.00–245.00)	205.00 (169.00–244.00)	.872
TG (mmol/L)	1.47 (1.07–1.99)	1.48 (1.07–2.01)	1.46 (1.07–1.99)	.949
TC (mmol/L)	4.21 (3.55–4.88)	4.20 (3.53–4.89)	4.21 (3.56–4.86)	.847
LDL (mmol/L)	2.81 (2.17–3.39)	2.80 (2.19–3.37)	2.83 (2.14–3.43)	.643
HDL (mmol/L)	1.04 (0.87–1.24)	1.03 (0.85–1.24)	1.04 (0.87–1.23)	.790
NEUT	4.08 (3.26–5.07)	4.00 (3.20–4.88)	4.22 (3.34–5.16)	.002
MO	0.48 (0.39–0.59)	0.47 (0.38–0.57)	0.49 (0.39–0.60)	.024
TLC	1.71 (1.34–2.11)	1.76 (1.39–2.18)	1.64 (1.29–2.05)	<.001
SIRI	1.25 (0.82–1.86)	1.13 (0.76–1.72)	1.37 (0.90–2.16)	<.001

Abbreviations: CE, cardioembolism; HDL, high‐density lipoprotein; LAA, large‐artery atherosclerosis; LDL, low‐density lipoprotein; NIHSS, National Institutes of Health Stroke Scale; SAO, small‐artery occlusion; SIRI, systemic inflammatory response index.; SOE, stroke of other determined etiology; SUE, stroke of undetermined etiology; TC, total cholesterol; TG, triglyceride; WBC, white blood cell.

**TABLE 2:** Multivariate regression analysis for risk factors of poststroke cognitive impairment (PSCI).

Variables	OR	95%CI	*p*‐Value
Age	1.048	1.036–1.060	<.001
Education	0.953	0.927–0.979	.001
Admission NIHSS scores	1.177	1.117–1.240	<.001
Gender	1.367	1.036–1.803	.027
Diabetes mellitus	1.445	1.114–1.874	.006
Smoking history	1.141	0.844–1.543	.393
Atrial fibrillation	1.195	0.731–1.952	.477
SIRI	1.226	1.095–1.373	<.001

Abbreviations: NIHSS, National Institutes of Health Stroke Scale; SIRI, systemic inflammatory response index.

## 3 RESULTS

### 3.1 Baseline characteristics between the PSCI and non‐PSCI groups

During the study period, 1342 eligible patients were included in the study. Among the 1342 patients, 690 were in the PSCI group, and 652 were in the non‐PSCI group. The distinctions between PSCI and non‐PSCI groups are provided in Table 1. The PSCI group showed significantly higher NEUT, MO, and SIRI levels than the non‐PSCI group (*p* < .001). Women, diabetes mellitus, atrial fibrillation, low TLC levels, and the older age group were more common among PSCI patients than among non‐PSCI patients (all *p* < .05). Additionally, there were also significant differences between the two groups in terms of years of education, smoking history and admission NIHSS scores (all *p* < .05).

### 3.2 Multivariable analysis for possible predictors of PSCI

In the logistic regression analysis, higher SIRI values were independently associated with the occurrence of PSCI in the acute phase of AIS, with an adjusted OR of 1.226 (95%CI: 1.095–1.373, *p* < .001) after adjusting for age, gender, education, admission NIHSS scores, diabetes mellitus, smoking history, and atrial fibrillation. Additionally, age (OR = 1.048, 95%CI: 1.036–1.060, *p* < .001), female (OR = 1.367, 95%CI: 1.036–1.803, *p* = .027), admission NIHSS scores (OR = 1.177, 95%CI: 1.117–1.240, *p* < .001), years of education (OR = 1.177, 95%CI: 1.117–1.240, *p* < .001), and diabetes mellitus (OR = 1.445, 95%CI: 1.114–1.874, *p* = .006) remained independently associated with the incidence of PSCI (Table 2).

### 3.3 Predictive model of early‐onset PSCI and model evaluation

**FIGURE 1:**
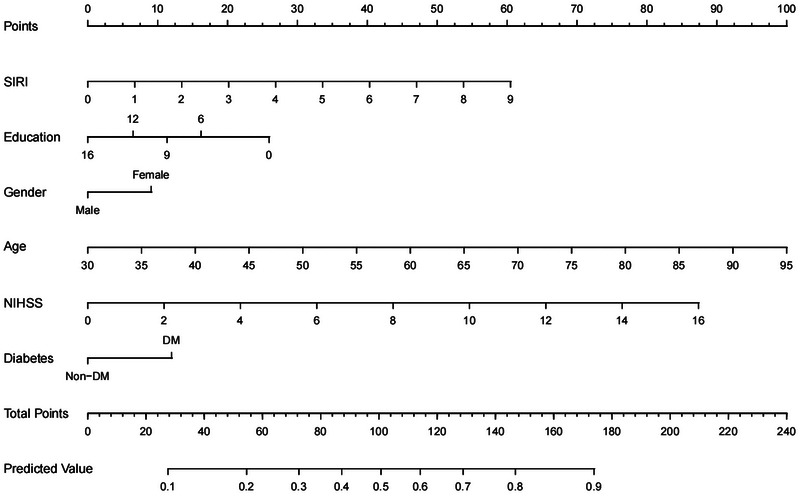
The nomogram for patients with poststroke cognitive impairment (PSCI).

**FIGURE 2:**
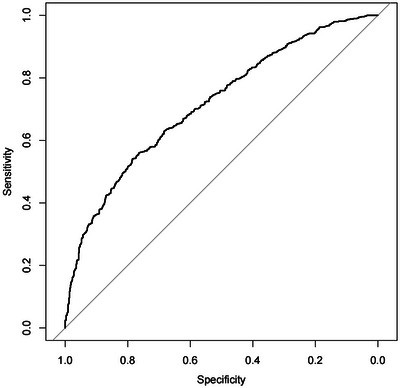
Receiver operating characteristic curve of the nomogram.

**FIGURE 3:**
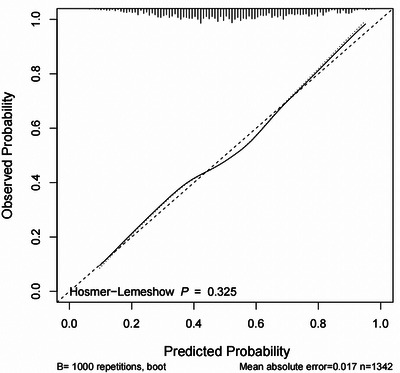
Calibration plot of the nomogram.

The nomogram was constructed by incorporating the six predictors according to the results of multivariable analysis (Figure 1). Each variable, as illustrated, has a corresponding score based on the results of performing the “rms” package in RStudio, and the sum of these values correlates to the probability of PSCI occurring on the probability axis. ROC curves were used to assess the discriminative ability of the nomogram. In our model, the AUC was 0.716, the sensitivity was 54%, and the specificity was 78%, demonstrating good discriminative ability (Figure 2). The calibration curve is a tool that is commonly used to assess consistency between a predicted situation and an actual one, using the method of 1000 bootstrap resamples. As shown in Figure 3, a good agreement was seen between the predicted risk and the observed risk in the calibration curves for this model. The Hosmer–Lemeshow test (*p* = .325) further confirmed the good calibration. Furthermore, DCA, which was carried out by calculating the net benefit at various threshold risks of the model using the package “rmda,” was used to assess the nomogram's clinical usefulness.

**FIGURE 4:**
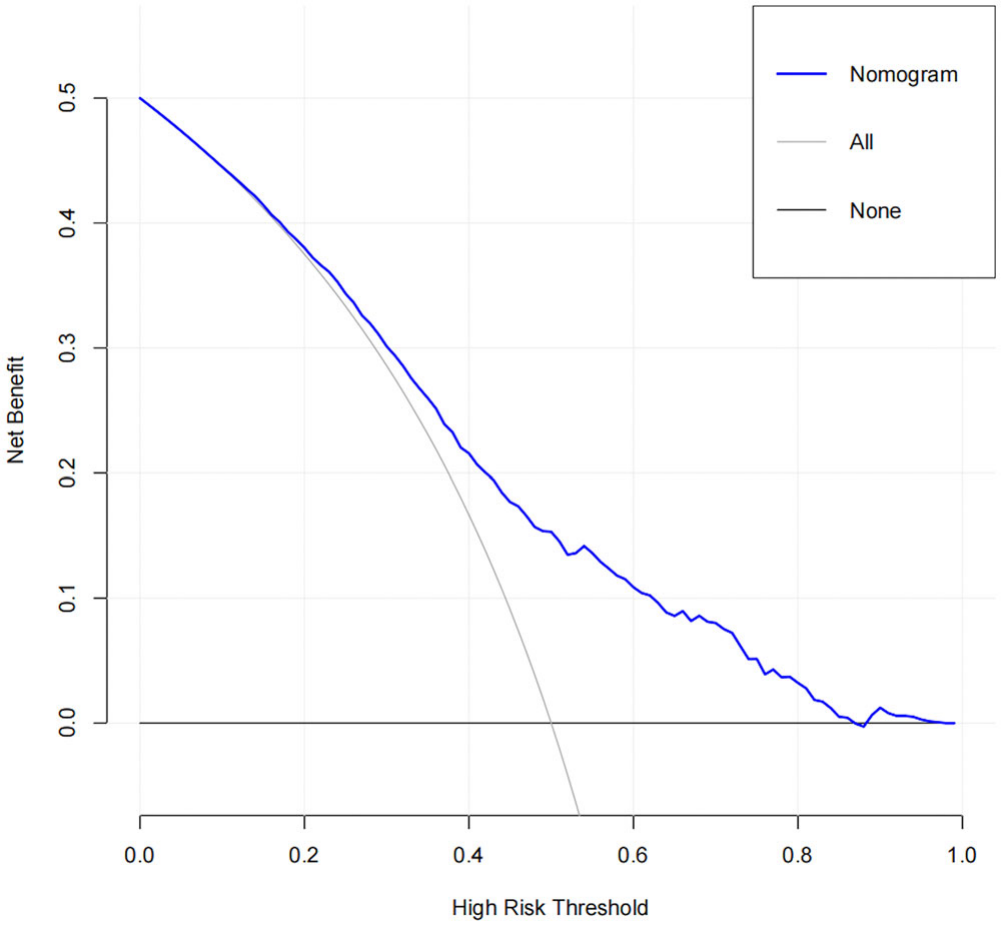
Decision curve analysis of the model.

Ultimately, when the threshold probability ranged from 0.2 to 0.9, the DCA model showed a positive net benefit, indicating that the DCA demonstrated the good clinical utility of the model (Figure 4).

## 4 DISCUSSION

Age, sex, the admission NIHSS score, diabetes mellitus, and educational level were thought to be related to the incidence of PSCI in previous research (Ding et al., 2019; Sun et al., 2014; Xu et al., 2023). To the best of our knowledge, there is currently no research that has examined the relationship between the easily obtainable peripheral inflammatory factors SIRI and PSCI. Our findings indicate that a higher SIRI at admission is associated with the occurrence of PSCI. In addition, this is the first study to construct a nomogram for the PSCI in the acute phase of AIS.

Several studies have already found an association between inflammatory factors and PSCI (El Husseini et al., 2020; Narasimhalu et al., 2015; Rothenburg et al., 2010; Wang et al., 2022). Nada et al. found that vascular cell adhesion molecule‐1, which functions as a pro‐inflammatory factor, plays a key role in WBC adhesion and migration and is associated with an increased risk of PSCI (El Husseini et al., 2020). Recently, a prospective study investigated the impact of IL‐6 on cognitive performance and showed elevated IL‐6 levels were independently associated with cognitive impairment in patients with ischemic stroke and transient ischemic attack (Wang et al., 2022). IL‐6 and IL‐8 are mainly produced by mononuclear macrophages in WBCs and are interleukin pro‐inflammatory factors (Mertowska et al., 2022). CRP is a cyclic pentamer protein that binds to the polysaccharide of *Streptococcus pneumoniae* capsule C and is formed by the aggregation of five identical subunits (23KD) in non‐covalent bonds. CRP is produced in hepatocytes under the transcriptional control of IL‐6 (Dufour, 2013). In addition, the three components of SIRI are all WBCs, so they are closely related. Moreover, despite the significant correlation between these inflammatory factors and cognitive function, these markers are rarely measured in clinical practice and usually require extra testing methods. Therefore, the SIRI was chosen as a target indicator for this study as an easily accessible and clinically applicable peripheral inflammatory indicator. Compared to the neutrophil‐to‐lymphocyte ratio, which was regarded as the conventional inflammatory marker, SIRI with a new component (monocytes related to the above inflammatory mediators) would reflect systemic inflammation better.

SIRI is a novel inflammatory index that includes neutrophils, monocytes, and lymphocytes from the peripheral blood. In recent years, SIRI has received extensive attention for its ability to predict outcomes in diseases like tumors, cardiovascular diseases, and infectious diseases (Ferrara et al., 2019; Hui et al., 2018; Pacheco‐Barcia et al., 2020). Moreover, elevated SIRI levels are associated with an increased risk of stroke as well as all‐cause mortality (Jin et al., 2021; Xia et al., 2023). Few studies have investigated the association between SIRI and cognitive impairment over the past few years (Liu et al., 2023; Wang et al., 2022). Wang et al. (2022) found that the elderly people with higher levels of SIRI may be at a greater risk of developing cognitive impairment. Similarly, another study on mild cognitive impairment in older adults found the same result (Liu et al., 2023). Based on the results of the above study, the present study also found that higher SIRI levels were independently associated with early‐onset PSCI.

There is no consensus on the precise mechanisms of PSCI because of its multifactorial pathophysiology. Hypoperfusion, oxidative stress, and inflammation, acting alone or in combination, are believed to affect the neurovascular unit and contribute to the pathogenesis of PSCI (Shang et al., 2022). A prospective study found that, irrespective of the size or location of the lesions, systemic inflammation significantly increases the risk of PSCI (Kliper et al., 2013). Previous search has discovered that lymphocyte counts in cognitive impairment patients are significantly lower than in healthy controls (Dong et al., 2019). In contrast to lymphocytes, neutrophils and monocytes increased in patients with cognitive dysfunction (Dong et al., 2019; Munawara et al., 2021). Moreover, the neutrophils, lymphocytes, and monocytes contained in SIRI play a vital role in the immune and inflammatory response (Germolec et al., 2018). A persistent inflammatory stimulation causes endothelial dysfunction, which disrupts the BBB. Animal models of endothelial dysfunction suggest that damage to the BBB disrupts the flow of oxygen and nutrients to the brain and allows toxins from around the brain to enter the central nervous system, triggering or exacerbating neurodegeneration, which can lead to PSCI (Bell & Zlokovic, 2009; Kisler et al., 2017). Therefore, the SIRI may be a very promising auxiliary diagnostic biomarker for PSCI.

The present study found that 51.6% of AIS patients present with cognitive impairment in the acute stage, which is in line with earlier studies (Sun et al., 2014; Xu et al., 2023). Age, admission NIHSS scores, and education level were also found to be associated with the occurrence of PSCI (Jacquin et al., 2014; Zha et al., 2021). As shown in the nomogram, higher age and NIHSS score play a greater weight in the prediction model than SIRI, suggesting that while paying attention to SIRI, older age and higher stroke severity often suggest a higher risk of poor prognosis. Furthermore, we found that patients with diabetes were more likely to suffer from PSCI, as was demonstrated in the previous study (Ding et al., 2019; Lee et al., 2023). In addition, we discovered that PSCI was more common among women than men. This phenomenon may be explained by the fact that older women have significantly higher levels of follicle‐stimulating hormone (FSH) than older men, and that the increased FSH binds to the FSH receptor on the surface of neurons and activates the C/EBPβ/AEP pathway, which in turn triggers an increase in Aβ and Tau pathology, leading to a greater risk of cognitive impairment in women (Xiong et al., 2022). Our results provide clinical data to support the subsequent analysis of risk factors for PSCI.

There are several limitations to this study. First, due to the cross‐sectional nature of the study, causality between SIRI and PSCI cannot be established. The explanation of our data is limited to the use of SIRI as a promising diagnostic and therapeutic target for PSCI in future investigations. Second, we did not include dynamic changes in SIRI levels. Admission SIRI combined with dynamic changes would be more detailed and could provide better predictive information. Finally, as PSCI can occur immediately following a stroke and last for a long period, cognitive function may differ in both the acute and chronic stages of stroke. The long‐term follow‐up evaluation of the cognitive function is therefore critical, other than within 2 weeks after stroke. A cognitive follow‐up was also performed on some patients by us 3 months, 6 months, and 1 year after stroke. However, due to the COVID‐19 outbreak during this period, many patients were unable to reach the hospital in time for follow‐up, so we need to further validate the correlation between SIRI and PSCI in future follow‐up studies. In spite of these limitations, we have demonstrated for the first time that SIRI is associated with the development of PSCI. Another strength of our study is the development of a nomogram for PSCI in the acute phase of AIS by using a certain number of data samples, which has never been done before. Clinically accessible indicators were chosen so that clinicians may easily determine whether a particular stroke patient is at risk for PSCI and take proper preventive measures.

## 5 CONCLUSION

Our study indicates that a higher SIRI may be correlated with the occurrence of PSCI, and the developed nomogram can be used to predict the risk of PSCI in stroke patients.

## AUTHOR CONTRIBUTIONS

**Min Chu**: Conceptualization; methodology; formal analysis; writing—original draft; data curation. **Yunhe Luo**: Conceptualization; methodology; formal analysis; writing—original draft; data curation. **Daosheng Wang**: Conceptualization; methodology; formal analysis; writing—original draft; data curation. **Zhuohang Liu**: Conceptualization; Validation. **Huicong Niu**: Conceptualization; validation. **Xuechun Wu**: Conceptualization; validation. **Yong Wang**: Conceptualization; validation. **Jixian Lin**: Conceptualization; writing—review and editing. **Qiang Wang**: Conceptualization; writing—review and editing. **Jing Zhao**: Conceptualization; writing—review and editing; investigation; funding acquisition; project administration; supervision.

## CONFLICT OF INTEREST STATEMENT

The authors declare no conflicts of interest.

### PEER REVIEW

The peer review history for this article is available at https://publons.com/publon/10.1002/brb3.3372.

## Data Availability

The data that support the findings of this study are available from the corresponding author upon reasonable request.
